# Enhanced Bioactivity of Fermented *Aralia cordata* Extract for Glucose and Immune Modulation

**DOI:** 10.3390/cimb47040294

**Published:** 2025-04-21

**Authors:** Heejong Shin, Hwapyung Kim, Gwangpyung Kim, Yikyoung Kim, Boyong Kim

**Affiliations:** 1 Department of Clinical Laboratory Sciences, College of Health Science, Korea University, Seoul 02841, Republic of Korea; hjshin0523@gmail.com; 2 Department of Plant Medicals, Andong National University, Andong 36729, Republic of Korea; kimhp031107@gmail.com; 3 Department of Biological Science, Andong National University, Andong 36729, Republic of Korea; kimgp050321@gmail.com; 4 Industry-Academic Cooperation Group, Catholic Sangji College, Andong 36686, Republic of Korea; radiorist@csj.ac.kr; 5 EVERBIO, 131, Jukhyeon-gil, Gwanghyewon-myeon, Jincheon-gun 27809, Republic of Korea

**Keywords:** *Aralia cordata*, glucose modulation, butyrate, L cell, intestinal cell

## Abstract

Excessive glucose absorption is a major contributing factor of metabolic disorders that necessitates effective therapeutic strategies. This study investigates the potential of fermented *Aralia cordata* extract (FACE) in regulating glucose transport and immune responses under high-glucose stress conditions. Caco-2 intestinal cells and L cells were treated with FACE to determine effects on key glucose-regulating proteins and cytokines. FACE treatment inhibited the expression of glucose transporters SGLT1 and GLUT2 while promoting GLP-1 secretion. This effect was associated with HDAC and somatostatin suppression, along with AMPK-γ upregulation. Notably, FACE inhibited DPP-4 expression, further enhancing GLP-1 stability and function. Immunomodulatory effects also occurred, specifically FACE promotion of T lymphocyte differentiation, with a stronger influence on Th2 cell development. Additionally, FACE increased the secretion of essential molecules for immune balance and inflammation control, including antimicrobial peptides LL-37 and defensin, along with cytokines IL-4 and IL-13. These findings suggest that FACE exerts dual effects of improving glucose regulation and modulating immune responses, highlighting its potential as a novel bioactive material for managing metabolic disorders and enhancing intestinal immunity. Further research is warranted to explore its clinical applicability in therapeutic formulations.

## 1. Introduction

Excessive sugar absorption has been increasingly linked to metabolic disorders, particularly through the dysregulation of key transporters and immune modulators. Sodium-glucose cotransporter 1 (SGLT1) and glucose transporter 2 (GLUT2) play pivotal roles in intestinal glucose uptake [1]. Overexpression or hyperactivity of these transporters can elevate blood glucose levels, contributing to insulin resistance and type 2 diabetes. Moreover, the chronic glucose overload may dampen the impact of incretins, such as glucagon-like peptide-1 (GLP-1), which regulates glucose homeostasis and stimulates insulin secretion [2]. Excessive glucose uptake also influences the expression of cathelicidin antimicrobial peptide LL-37, critical for maintaining intestinal barrier integrity and modulating inflammation. Alterations in LL-37 expression impair host defense mechanisms, increasing susceptibility to infections and chronic inflammation [3]. Immune cell functions, particularly phagocytosis, are also affected, as hyperglycemia-induced oxidative stress can impair macrophage activity and reduce pathogen clearance [4]. The dangers of combined metabolic and immune dysregulation underscore the need to explore therapeutic targets for mitigating the effects of excessive sugar absorption.

*Aralia cordata*, commonly known as udo in East Asia, is a perennial herbaceous plant traditionally used in herbal medicine [5]. Its extract is rich in bioactive compounds with antioxidant, anti-inflammatory, and antimicrobial properties, including saponins, flavonoids, and phenolic acids [5]. These diverse biological functions have led to a wide study of *Aralia cordata* extract (ACE) [6].

Short-chain fatty acids (SCFAs), particularly butyric and propionic acids, are essential metabolites in glucose regulation and gut health. They are primarily produced during dietary fiber fermentation by gut microbiota, but lactic acid bacteria (LAB) fermentation can significantly influence their concentration and bioactivity. Recent studies have shown that SCFAs contribute to the metabolic benefits of LAB strains, such as *Lactobacillus plantarum* and *Bifidobacterium bifidum* (phylum Actinobacteria), which enhance butyric and propionic acid production during fermentation [7]. Specifically, LAB fermentation facilitates polysaccharide breakdown and stimulates cross-feeding mechanisms between bacteria. Substrates from LAB-induced degradation of complex carbohydrates are then further metabolized into SCFAs by other commensal microbes [8]. For instance, during the fermentation of prebiotic-enriched substrates, LAB increases, while butyric acid yield strengthens the intestinal barrier and mitigates inflammation. Additionally, LAB upregulation of propionic acid reduces hepatic gluconeogenesis and modulates lipid metabolism [9]. The LAB-mediated optimization of the butyric acid/propionic acid ratio appears to enhance their synergistic effects on glucose absorption and insulin sensitivity [9].

Several studies have demonstrated the potential of ACE in modulating glucose metabolism, particularly increasing insulin sensitivity and lowering hyperglycemia [10]. However, limited evidence exists regarding ACE’s specific effects on glucose transporters critical for intestinal glucose absorption, such as SGLT-1 and GLUT2 [1]. Additionally, no studies have explored the role of ACE in regulating GLP-1, a key incretin hormone that enhances insulin secretion and reduces postprandial glucose levels. Given the growing interest in natural compounds that influence glucose-regulating pathways, investigating the impact of ACE on SGLT-1, GLUT2, and GLP-1 is crucial. Moreover, identifying the molecular targets of ACE could uncover new therapeutic opportunities for managing type 2 diabetes and other metabolic disorders.

This study aimed to evaluate the effects of fermented ACE (FACE) on glucose absorption regulation through pathways involving SGLT-1, GLUT2, and GLP-1. We also investigated the compound’s potential modulation of intestinal immune response. By uncovering the potential of FACE in regulating these critical pathways, our research seeks to offer a novel functional biomaterial and contribute to advancing plant-derived therapeutic agents for metabolic health.

## 2. Materials and Methods

### 2.1. Extraction and Fermentation

Whole A. cordata (Jincheon, Chungcheongbuk-do, Republic of Korea) were dried, ground, and extracted with 70% methanol (Sigma-Aldrich, St. Louis, MO, USA) using a Soxhlet apparatus, then sonicated for 60 min at 60 Hz and 25 °C (Sungdong, SD-D200H, Seoul, Republic of Korea). The extract was filtered through Whatman No. 1 filter papers (Cytiva, Marlborough, MA, USA), concentrated under reduced pressure using a rotary evaporator (Buchi, Flawil, Switzerland), and freeze-dried using a lyophilizer (Labconco, Kansas City, MO, USA). Next, ACE was prepared by mixing 10 g of crude extract with 90 mL of sterilized water containing 5% (*w*/*v*) glucose (Sigma-Aldrich). The mixture was sterilized at 121 °C for 15 min, cooled to room temperature, and inoculated with 1 × 10^8^ CFU/mL of Lactiplantibacillus plantarum KCTC 3104 (Jeongeup, Republic of Korea). Fermentation was conducted in an anaerobic bioreactor (Bioflo 120, Eppendorf, Hamburg, Germany) for 36 h at 37 °C and pH 6.0 (maintained using 1 N NaOH and 1 N HCl, Thermo Fisher Scientific, Waltham, MA, USA). After fermentation, the broth was centrifuged at 10,000× *g* for 15 min using a high-speed centrifuge (Avanti J-26XP, Beckman Coulter, Brea, CA, USA), and the supernatant was collected using a 0.2 μm syringe filter (Sartorius AG, Göttingen, Germany).

### 2.2. Analysis of Butyrate in FACE and ACE

Butyrate content in the fermented samples (ACE, FACE) was measured using the Butyrate ELISA Kit (Abcam, Cambridge, MA, USA) and evaluated with a microplate reader (AMR-100; Allsheng, Hangzhou, China). Butyrate was quantified using high-performance liquid chromatography (HPLC) with a standard curve constructed from butyric acid (≥98% purity; Sigma-Aldrich, St. Louis, MO, USA). After filtering FACE and ACE using 0.22 μm syringe filters, the two fermented products were diluted with ultrapure water prepared using a Milli-Q water purification system (Millipore, Burlington, MA, USA). HPLC analysis was performed on a Prominence HPLC system equipped with a refractive index detector (RID; Shimadzu, Kyoto, Japan) using a ZORBAX Eclipse Plus C18 column (4.6 × 250 mm, 5 μm; Agilent Technologies, Santa Clara, CA, USA). The mobile phase consisted of 0.1% (*v*/*v*) phosphoric acid in ultrapure water and acetonitrile (both HPLC grade; Merck, Darmstadt, Germany), delivered at a flow rate of 1.0 mL/min. The column temperature was maintained at 35 °C, and the injection volume was set to 20 μL. Retention time for butyrate was approximately 2.8–3.0 min. Peak areas from standard solutions (5–50 mg/L) were used to construct the calibration curve. Chromatographic data were acquired and processed using LabSolutions ver. 5.97 (Shimadzu, Kyoto, Japan).

### 2.3. Cell Culture and Establishment of Treatment Dosages

Cultured Caco-2 cells (ATCC, Manassas, VA, USA) and macrophages were exposed to various ACE and FACE concentrations (0–5 mg/mL) for 1 day. To determine appropriate ACE and FACE dosages, exposed cells were evaluated for cytotoxicity using PI-Annexin V (Thermo Fisher Scientific), a flow cytometer (BD FACSCalibur, BD Biosciences, Franklin Lakes, NJ, USA), and FlowJo 10.8.1 (BD Biosciences). Except for the GLP-1 assay and immunocytochemistry, all other experiments exposed cells to four conditions under the following time settings: 1 day of Control (Con, glucose 1.0 g/L of 5.5 mM), FACE (1.0 g/L of 5.5 mM), and high glucose (HG, 4.5 g/L of 25 mM); 1 day of FACE, followed by an additional day of HG (FACE+HG). For the GLP-1 assay and immunocytochemistry, FACE+HG was administered as a combined treatment for 10 days.

### 2.4. Enzyme-Linked Immunosorbent Assay

**Figure 1:**
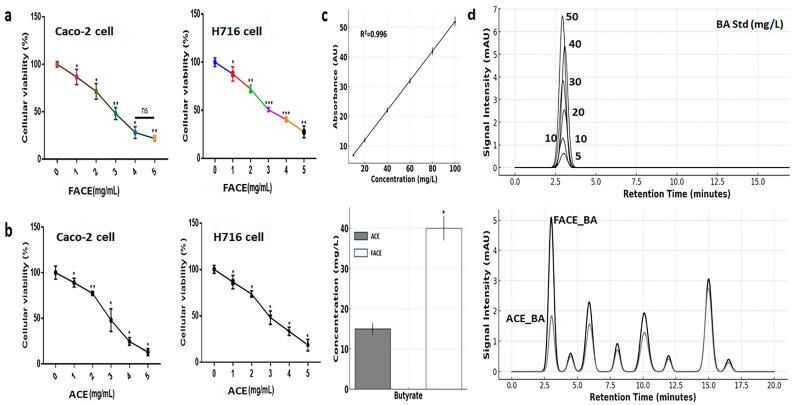
Evaluation of active compounds and cytotoxicity for ACE and FACE. (**a**,**b**) Cytotoxicity test of FACE and ACE in intestinal cells (Caco-2) and L cells (H716 cells). (**c**,**d**) ELISA and HPLC results on butyric acid in FACE and ACE. ACE, *Aralia cordata* extract; FACE, fermented *Aralia cordata* extract; BA, butyrate; Std, standard; ns, not significant (* *p* < 0.05, ** *p* < 0.01, *** *p* < 0.001).

The intestinal L cell line NCI-H716 (ATCC, Manassas, VA, USA) was cultured and exposed to four different conditions: Con, HG, FACE, and FACE+HG. The amount of GLP-1 (Invitrogen, Thermo Fisher Scientific, Waltham, MA, USA) in the culture medium was measured on days 5 and 10 after exposure. LL-37 (Novus Biologicals, Bio-Techne, Minneapolis, MN, USA) and defensin (human beta-defensin 1, Thermo Fisher Scientific) were measured after exposing Caco-2 cells to the four experimental conditions (Con, HG, FACE, and FACE+HG), as well as to various FACE and ACE concentrations for 1 day. To investigate FACE regulation of GLP-1 activity, somatostatin (Thermo Fisher Scientific) and DPP-4 (Dipeptidyl Peptidase-4) protein (Thermo Fisher Scientific) secretion were measured in Caco-2 cells exposed to five different conditions: Con, HG, High Butyrate (HB; 1 mM/mL, Sigma-Aldrich), FACE, and FACE+HG. Additionally, to assess FACE promotion of IL-4 (Thermo Fisher Scientific) and IL-13 (Thermo Fisher Scientific) secretion in T lymphocytes, Jurkat T cells (ATCC) were cultured in Dulbecco’s modified Eagle medium (DMEM) with 100 μL of supernatant and conditioned media (CM) from the four treatment conditions (CCM, HGCM, FACECM, and FACECM+HGCM) for 1 day. Based on the results of preliminary dose determination experiments (Figure 1 and Figure S1), all cell types were treated with 3 mg/mL of either FACE or ACE. Cytokine secretion levels were then measured using a microplate reader (AMR-100; Allsheng, Hangzhou, China).

### 2.5. Flow Cytometry

After Caco-2 cells were cultured under various conditions (Con, HG, FACE, and FACE+HG) for 1 day, they were fixed with 2% paraformaldehyde for 8 h. To investigate butyrate’s effects on expression of key intracellular markers (HDAC, histone deacetylase; AMPK-γ, AMP-activated protein kinase gamma; T-bet, T-box transcription factor TBX21; GATA3, GATA binding protein 3) involved in glucose metabolism and immune regulation, Caco-2 cells were exposed to five different conditions (Con, HG, HB, FACE, and FACE+HG). After treatment with the Fc receptor blocker reagent (BD Biosciences), cells were incubated with the FITC-GLUT2 antibody (Thermo Fisher Scientific), FITC-SGLT1 (Thermo Fisher Scientific), FITC-anti-HDAC1 (Abcam), AMPK-γ (Abcam) conjugated with the APC labeling kit (EasyLink Fluorescent Labeling Kits, Abcam), FITC-anti-T-bet (Abcam), and APC-anti-GATA3 (Abcam) for 1 day. Prior to antibody treatment for intracellular analysis, cells were pretreated with 0.02% Tween 20 (Sigma-Aldrich) for 30 min, then treated with 3 mg/mL FACE and ACE (Figure 1 and Figure S1). Stained cells were evaluated using a flow cytometer (BD FACScalibur), FlowJo 10.6.1 (BD Science), and Prism 7 (GraphPad, San Diego, CA, USA).

### 2.6. Immunocytochemistry

After Caco-2 cells were cultured under various conditions (Con, HG, FACE, and FACE+HG) for 10 days, they were fixed with 2% paraformaldehyde for 12 h, pretreated with Fc receptor blocker (BD Bioscience), and stained for 1 day using the FITC-GLUT2 antibody (Thermo Fisher Scientific). Stained cells were evaluated using a fluorescence microscope (Eclipse Ts-2, Nikon, Shinagawa, Japan) and counted in NIS-elementsV5.11 (Nikon).

### 2.7. Evaluation of Glucose Uptake

To assess the effect of FACE exposure on glucose uptake, Caco-2 cells exposed to the four experimental conditions were incubated with fluorescently labeled glucose using a glucose uptake assay reagent (Cayman Chemical Company, Ann Arbor, MI, USA). To ensure proper assay conditions, glucose-free DMEM (Thermo Fisher Scientific) was used. Cells were then treated with 3 mg/mL of FACE and ACE before evaluation using a flow cytometer (BD FACScalibur), FlowJo 10.8.1 (BD Bioscience), and Prism 7 (GraphPad, San Diego, CA, USA).

### 2.8. Evaluation of Glucose Absorption In Vivo

Brine shrimp (*A. franciscana*, TTO TTO-USA Co., San Francisco, CA, USA) cysts were developed in artificial seawater at 30 °C (pH 8.0) [11,12] for 2 days. Meta-nauplii were sorted from nauplii based on negative phototaxis behavior, confirmed using a microscope (Eclipse Ts-2, Nikon, Shinagawa, Japan). Isolated nauplii split into four conditions (Con; HG, 450 μg/mL; FACE, 3 mg/mL; FACE+HG) were treated with 10 mg/mL FITC-labeled glucose (Cayman Chemical Company) for 48 h. Next, the effect of FACE was evaluated under a fluorescence microscope (Eclipse Ts-2, Nikon, Shinagawa, Japan) and NIS-elements V5.11 (Nikon). Stained cells in nauplii were counted manually.

### 2.9. Statistics

All in vitro and clinical experiments (three independent replicates; five measurements per individual per measurement day) were analyzed using one-way ANOVA with post hoc analysis (Scheffe’s method) in Prism 7 (GraphPad). Before conducting ANOVA, data normality was assessed using the Shapiro–Wilk test.

## 3. Results

### 3.1. Treatment Dosages and Butyrate Content in FACE and ACE

The results of cytotoxicity evaluations revealed no differences between FACE and ACE, and cytotoxicity was low for both (Figure 1a,b), with the final exposure concentration being set to 3 mg/mL (Figure 1 and Figure S1). Interestingly, as a result of fermentation at a continuous pH of 6.0, FACE contained approximately 2.7 times more butyrate than ACE (Figure 1c,d). Results from HPLC and ELISA analyses were very similar (Figure 1c,d), indicating that the average butyric acid concentration in FACE was approximately 40 mg/L, corresponding to about 0.454 mM (Figure 1).

### 3.2. Comparison of Potential for Glucose and Immune Modulation Between FACE and ACE

**Figure 2:**
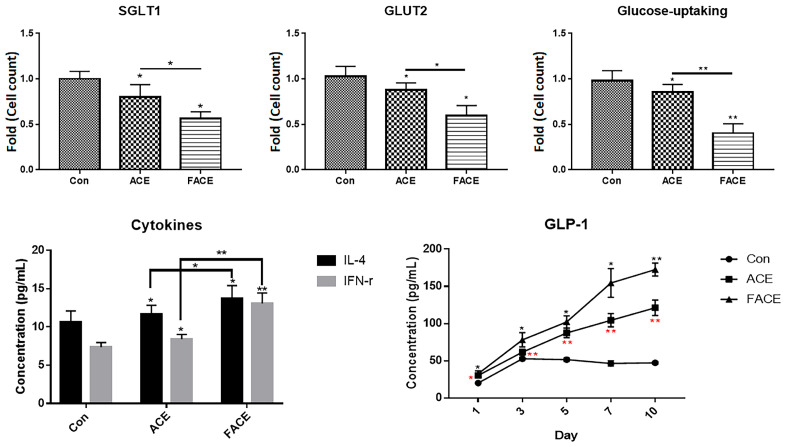
Comparison of FACE and ACE effects on glucose absorption regulation and immune activity. Caco-2 cells were used to evaluate SGLT1 and glucose uptake, as well as GLUT2 expression. GLP-1 expression and cytokine secretion regulation were evaluated using L cells (H716 cells) and T cells, respectively. SGLT1 and GLUT2 expressions were compared using flow cytometry, whereas GLP-1 and cytokine expressions and secretions were assessed with ELISA. Red asterisks indicate the *p*-values of ACE compared with the control group. Con, control; FACE, fermented *Aralia cordata* extract; ACE, *Aralia cordata* extract; ns, not significant (* *p* < 0.05, ** *p* < 0.01).

A comparative evaluation of FACE and ACE revealed that FACE exhibited enhanced regulatory effects on glucose absorption and immune activity (Figure 2). Compared with ACE, FACE was superior in inhibiting glucose absorption in intestinal cells and suppressing blood glucose elevation. This was achieved through the inhibition of SGLT expression, suppression of GLUT2 expression, and promotion of GLP-1 expression (Figure 2). Particularly encouraging was the FACE-induced increase and sustained high concentration of GLP-1, as this effect influences both glucose regulation and immune activity (Figure 2).

### 3.3. Glucose Regulation Potential of FACE

**Figure 3:**
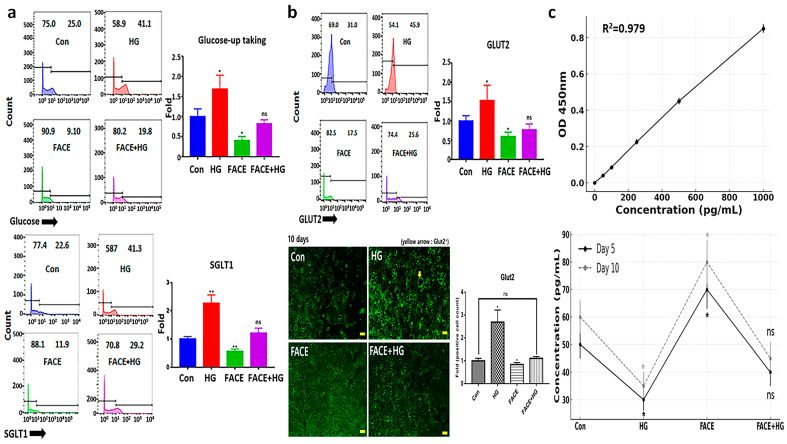
Glucose regulation potential of FACE. (**a**) Glucose uptake and expression of SGLT1 in intestinal cells exposed to various conditions. b GLUT2 expression in intestinal cells under various conditions. (**b**) Stained cells with FITC-GLUT2 antibodies in intestinal cells exposed to various conditions. (**c**) Levels of GLP-1 secreted from intestinal cells under various conditions for 10 days. Con, control; HG, high glucose exposure; FACE, fermented *Aralia cordata* extract; FACE+HG, HG after exposing to FACE; ns, not significant (* *p* < 0.05, ** *p* < 0.01) (Scale bars = 20 μm).

Exposure to HG increased the expression of the intestinal glucose transporter SGLT1 by 2.3 times (Figure 3a). Treatment with FACE reduced SGLT1 expression by 1.6 times (Figure 3a). Intestinal cells stimulated with FACE+HG upregulated SGLT1 by 1.2 times; compared with the HG condition, this represented a 1.9-times decrease in SGLT1 expression (Figure 3a). Similar results were observed using flow cytometry to measure actual effects on glucose uptake in cells (Figure 3).

Treatment with HG caused a 1.5-times upregulation of GLUT2, responsible for transporting glucose from intestinal cells to the bloodstream (Figure 3b). In contrast, FACE treatment suppressed GLUT2 expression, suggesting its potential to inhibit blood glucose elevation (Figure 3b). Under FACE+HG, GLUT2 expression was 1.5 times lower than under HG alone, demonstrating the potential of FACE as a bioactive material for blood glucose regulation (Figure 3a,b). Additionally, after 10 days of exposure, immunocytochemistry results showed similar trends (Figure 3b). Particularly, L cell expression of GLP-1 was approximately 2.2 times higher on both days 5 and 10 of FACE+HG exposure compared to HG exposure. Under HG stress, GLP-1 secretion in L cells decreased by approximately 40% (Figure 3c). However, the protective effect of FACE maintained secretion at levels similar to the control group, despite exposure to HG (Figure 3c). In summary, the FACE+HG group did not differ significantly from the control group, but did differ significantly from the HG group.

**Figure 4:**
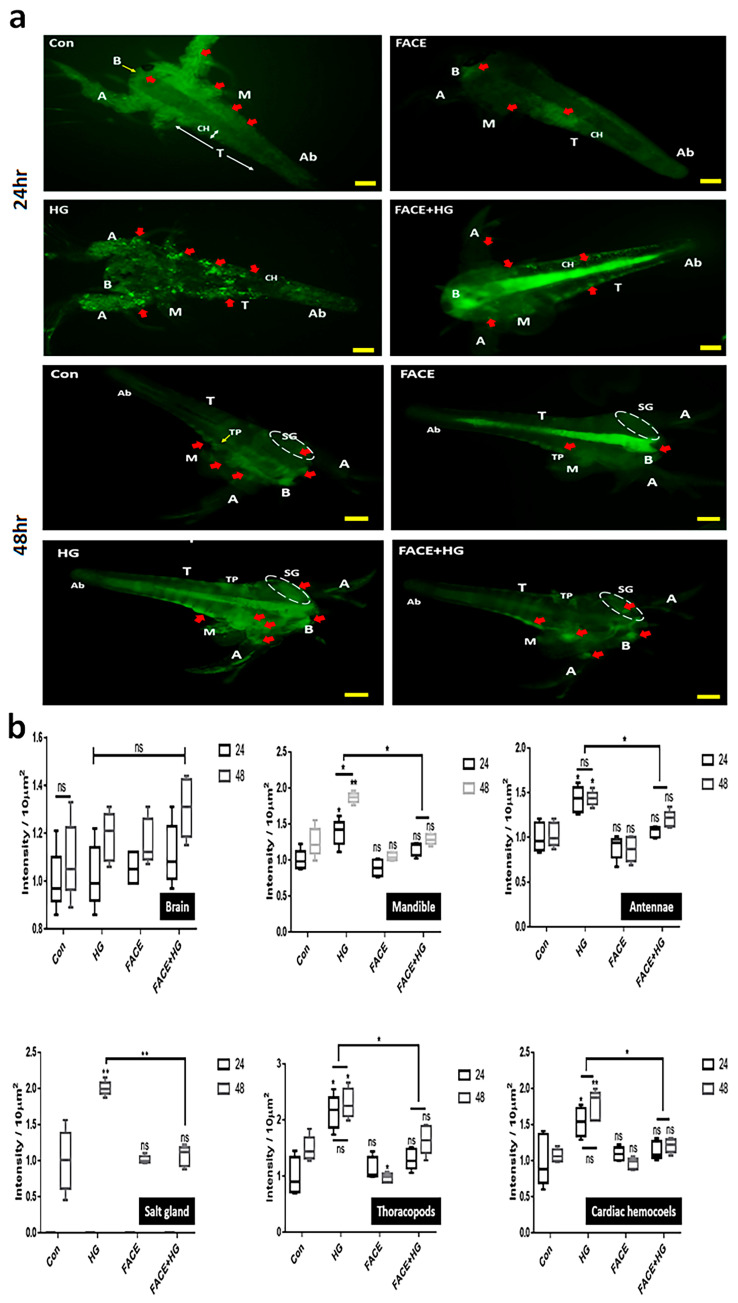
FACE modulation of glucose absorption in *Artemia franciscana*. (**a**) Images for up-taken FITC-glucose in midgut (MG) and cardiac hemocoels (CH) of *A. franciscana* meta-nauplii under the four conditions (Con, HG, FACE, and FACE+HG). (**b**) Fluorescence intensities for absorbed glucose in *A. franciscana* under the four conditions. Con, control; HG, high glucose; FACE, fermented *Aralia cordata* extract; FACE+HG, HG after 14 h exposure to FACE; A, antennae; B, brain; SG, salt gland; M, mandible; TP, thoracopods; T, thorax; Ab, abdomen; red arrows, glucose positive regions; ns, not significant (* *p* < 0.05, ** *p* < 0.01) (bar scale = 50 μm).

We next measured glucose uptake using meta-nauplii of the zooplankton *A. franciscana*. Fluorescent intensities across FACE+HG and HG treatments indicated that FACE addition inhibited glucose uptake and accumulation in meta-nauplii exposed to glucose for 24 h. This inhibitory effect was observed in all tissues except the brain, with an average reduction of 46% from levels under HG exposure (Figure 4). After 48 h of exposure, glucose accumulation decreased by 58% on average (Figure 4).

### 3.4. Mechanism Underlying FACE Regulation of Intracellular Glucose and Immune Response

**Figure 5:**
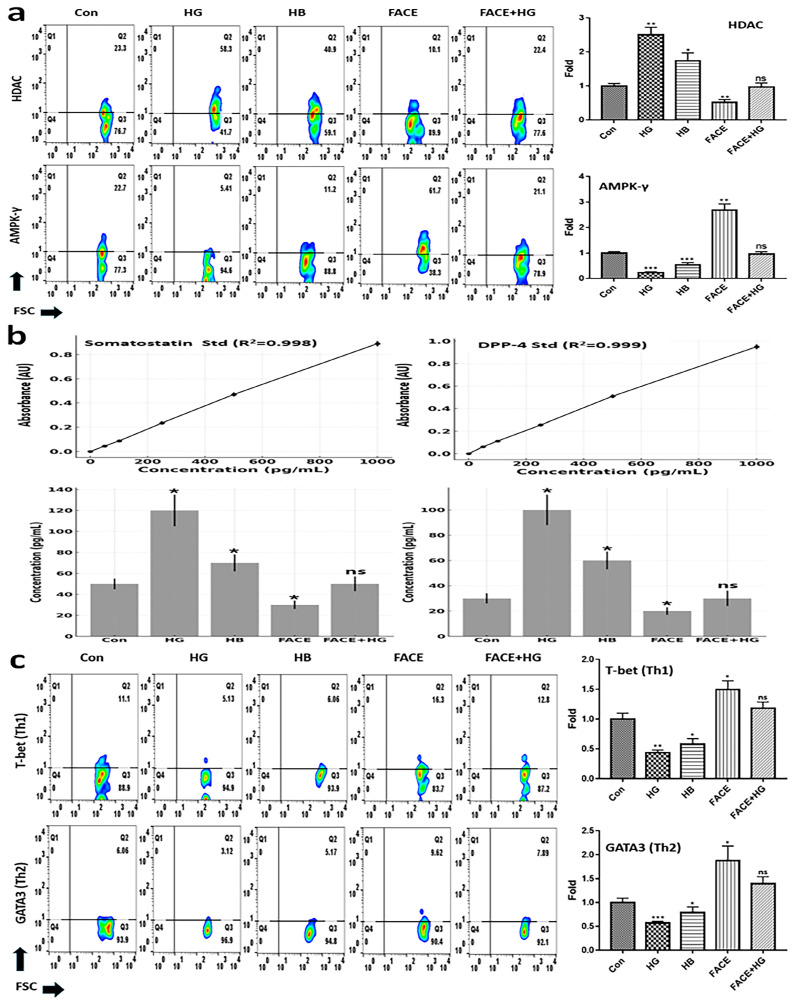
Effects of FACE on glucose modulation and T lymphocyte differentiation. (**a**) Expression of HDAC and AMPK-γ, key markers for glucose modulation, in intestinal cells under various conditions. (**b**) Expression of proteins involved in promoting and blocking GLP-1 synthesis. (**c**) FACE-induced T lymphocyte differentiation into Th1 and Th2 cells. Con, control; HG, high glucose exposure; HB, high butyrate (10 mM/mL); FACE, fermented *Aralia cordata* extract; FACE+HG, HG after exposure to FACE; ns, not significant (* *p* < 0.05, ** *p* < 0.01, *** *p* < 0.001).

To investigate the mechanism of SGLT1 and GLUT2 downregulation in intestinal epithelial cells exposed to FACE, we examined their regulatory enzymes HDAC and AMPK-γ. The results showed that FACE suppressed HDAC expression while promoting AMPK-γ expression (Figure 5a). Furthermore, the effect of high butyrate concentrations was similar to the effects of HG exposure (Figure 5a). However, the addition of FACE after HG exposure resulted in a maintenance of HDAC and AMPK-γ expression at levels comparable to control (Figure 5a). Interestingly, FACE inhibited DPP-4 secretion in intestinal epithelial cells, thereby promoting GLP-1 synthesis and maintenance (Figure 5b).

Next, we found that FACE enhanced lymphocyte differentiation into Th1 and Th2 cells, particularly promoting Th2 proliferation (Figure 5c). Differentiation into Th1 and Th2 cells was 3.12 and 2.28 times higher, respectively, than the levels seen under HG exposure alone (Figure 5c). A high butyrate concentration (10 mM) inhibited glucose regulation and immune cell differentiation in intestinal cells, similar to the effects observed under HG exposure. However, when treated with FACE (3 mg/mL) along with an optimal butyrate concentration (0.454 mM), glucose regulation and immune cell differentiation were promoted (Figure 5). Consistent with other results (Figure 3 and Figure 4), FACE+HG did not differ significantly from the control group, but did differ significantly from the HG group.

### 3.5. Evaluation of FACE Immune Modulation

**Figure 6:**
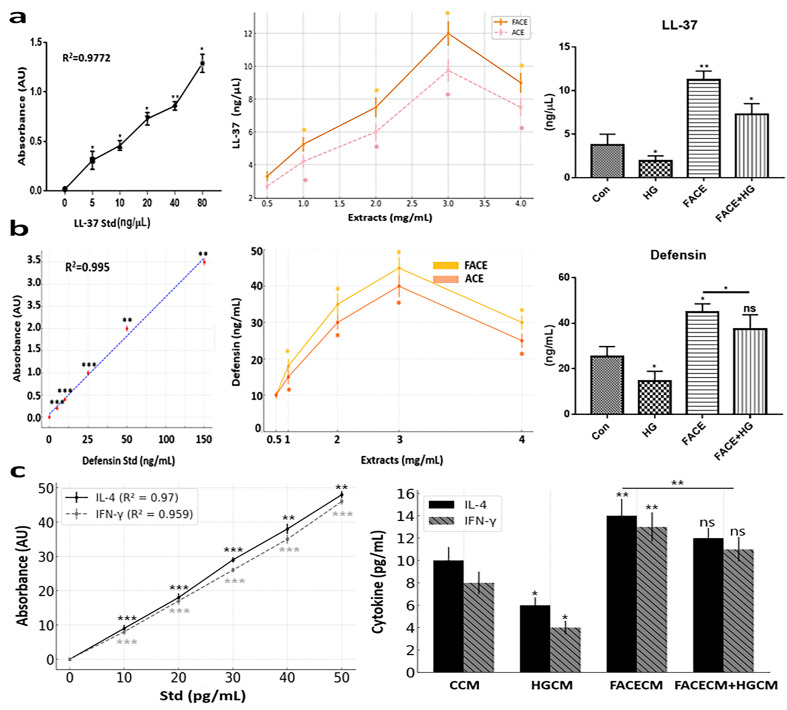
Effect of FACE on immune modulation. (**a**) ELISA of LL-37 secreted from intestinal cells under various conditions. (**b**) Defensin expression in intestinal cells exposed to various FACE and ACE concentrations. (**c**) IL-4 and IL-13 expression in T cells under four conditions. CCM, control-conditioned media; HGCM, high glucose exposure-conditioned media; FACECM, fermented *Aralia cordata* extract-conditioned media; FACECM+HGCM, HGCM after exposure to FACECM; Con, control; HG, high glucose exposure; FACE, fermented *Aralia cordata* extract; FACE+HG, HG after exposure to FACE; Std, standard; ns, not significant (* *p* < 0.05, ** *p* < 0.01, *** *p* < 0.001).

After comparing outcomes across treatments (Con, HG, FACE, and FACE+HG), we found that HG conditions suppressed both LL37 and defensin synthesis (Figure 6a,b). In contrast, FACE treatment increased LL37 and defensin synthesis by 2.3 and 2.5 times, respectively, from HG treatment levels (Figure 6a,b). Additionally, FACE increased LL37 and defensin synthesis by 22% and 20%, respectively, from ACE levels (Figure 6a,b). Furthermore, in T lymphocytes exposed to conditioned media, FACE exposure after HG exposure increased IL-4 and IL-13 secretion, respectively, by 3 and 2.8 times the levels observed under HG exposure alone (Figure 6c). As before (Figure 5), the FACE+HG group was comparable to the control group but significantly different from the HG group.

## 4. Discussion

The development of natural bioactive materials to regulate blood glucose absorption is essential for addressing the rising prevalence of diabetes and other metabolic disorders. Natural compounds, such as polyphenols and saponins, have considerable potential in modulating glucose metabolism and enhancing insulin sensitivity [13]. Recent studies have highlighted the need for further investigation into plant-derived bioactive compounds that can inhibit α-glucosidase and α-amylase activity, thereby reducing postprandial glucose levels [14]. Additionally, the safety and efficacy of these natural materials must be rigorously evaluated to ensure their clinical applicability [15]. Therefore, continued research on natural bioactive substances for blood glucose regulation is crucial for developing novel therapeutic strategies.

This study elucidates the promise of FACE as a functional biomaterial for regulating blood glucose levels and modulating intestinal immune responses. Compared with AC, FACE induced a greater increase in butyrate. Butyric acid plays a crucial role in enhancing insulin sensitivity and reducing glucose absorption, promoting gut barrier integrity and modulating intestinal hormones such as GLP-1 [16]. Butyric acid produced by gut microbiota plays a crucial role in maintaining immune homeostasis and has been associated with anti-inflammatory effects [17]. These findings suggest that FACE has major potential as a blood glucose regulator and an enhancer of intestinal immune activity. Our key findings demonstrated that FACE significantly downregulated glucose transporters SGLT1 and GLUT2 in vitro and in vivo, thereby decreasing glucose uptake. This function is crucial, given the association between glucose transporter dysregulation and metabolic disorders, particularly type 2 diabetes. By inhibiting the expression of these transporters, FACE can contribute to alleviating insulin resistance, a hallmark of type 2 diabetes. Additionally, SGLT1 and GLUT2 dysregulation is associated with metabolic diseases beyond type 2 diabetes. Abnormal activity of SGLT1 can contribute to conditions such as intestinal glucose-galactose malabsorption, characterized by severe diarrhea and dehydration [18]. Additionally, SGLT1 overexpression has been implicated in certain cancers, where increased glucose uptake faciliates rapid tumor growth [19]. GLUT2 dysregulation is linked to Fanconi–Bickel syndrome, a rare glycogen storage disorder that leads to hepatomegaly, hypoglycemia, and growth retardation [20]. Moreover, altered GLUT2 function is associated with non-alcoholic fatty liver disease and metabolic syndrome, conditions marked by insulin resistance and lipid accumulation in the liver [21].

In this study, FACE inhibited SGLT1 and GLUT2 expression in intestinal cells while promoting GLP-1 secretion in L cells. To investigate the underlying mechanisms of these effects, we evaluated the expression of HDAC, AMPK-r, somatostatin, and DPP-4 in intestinal cells (Figure 5). Interestingly, FACE suppression of HDAC and upregulation of AMPK-γ played a crucial role in inhibiting SGLT1 and GLUT2 expression. Moreover, these changes were associated with somatostatin and DPP-4 downregulation, which ultimately enhanced GLP-1 expression and secretion from L cells (Figure 3 and Figure 5). HDAC plays a key role in chromatin remodeling and gene expression; its inhibition improves insulin sensitivity and reduces glucose transporter expression, contributing to lower blood glucose levels [22]. AMPK-γ functions as an energy sensor that enhances glucose uptake by promoting GLUT4 translocation and suppressing gluconeogenesis [23]. Somatostatin, a hormone that inhibits insulin, glucagon, and GLP-1 secretion, negatively impacts glucose regulation when elevated [24]. DPP-4 rapidly degrades incretin hormones like GLP-1, and its inhibition is widely used to improve insulin secretion and glycemic control [25]. The sustained promotion of these markers plays an important role in maintaining high GLP-1 concentration and activity, indicating that FACE significantly contributes to its prolonged expression and continuous activation. GLP-1 itself is a potent incretin that enhances insulin release, suppresses glucagon secretion, and delays gastric emptying, playing a crucial role in postprandial glucose regulation [2].

Furthermore, FACE exhibited immunomodulatory properties, specifically enhancing GLP-1 secretion, upregulating antimicrobial peptides such as LL-37 and defensin, and promoting cytokine (IL-4 and IL-13) secretion from T lymphocytes. Notably, FACE promoted T lymphocyte differentiation into both Th1 and Th2 cells, with a particularly enhanced effect on Th2 differentiation. The upregulation of LL-37 and defensins enhances the activation of T lymphocytes, increasing IL-4 and IL-13 secretion [26]. These cytokines play a pivotal role in promoting anti-inflammatory responses and regulating immune homeostasis [26]. This mechanism highlights the interplay between antimicrobial peptides and adaptive immunity in maintaining mucosal defense and metabolic balance [26]. In particular, T cell secretion of IL-4 and IL-13 significantly influences glucose metabolism and immune regulation within the intestinal environment [27]. Primarily produced by Th2 cells, IL-4 promotes anti-inflammatory responses and has been implicated in enhancing insulin sensitivity via inducing M2 macrophage polarization, which is associated with improved metabolic outcomes [28]. IL-13 is a crucial regulator of glucose absorption in the small intestine, modulating the expression of glucose transporters such as SGLT1 and GLUT2. IL-13 also promotes M2 macrophage polarization and modulates intestinal epithelial barrier function [29]. Further investigation is required to elucidate the therapeutic potential of IL-13 in metabolic and inflammatory diseases. Nevertheless, the balance between IL-4 and IL-13 secretion is crucial for maintaining metabolic homeostasis and immune regulation in the gut. Therefore, therapeutic strategies targeting the modulation of these cytokines may offer potential benefits in managing conditions such as type 2 diabetes and inflammatory bowel disease.

The composition of ACE likely includes a variety of phytochemicals (e.g., saponins, polyphenols, flavonoids, and terpenoids) that contribute to its antioxidant and anti-inflammatory effects [30]. Fermentation of ACE appears to enhance its biological activity; FACE may additionally contain microbial metabolites, including organic acids, peptides, and bioactive enzymes. Consequently, FACE is more effective and functionally competitive than ACE, possessing dual functionality in glucose and immune regulation. This versatility makes FACE suitable as a material in various industrial applications, including health foods, functional foods, and pet treats. However, one limitation of this study is that in vivo experiments were conducted using *Artemia franciscana* meta-nauplii as an alternative model, which may not fully represent mammalian metabolic pathways.

## 5. Conclusions

Our findings highlight the major potential of FACE as a functional biomaterial for blood glucose regulation and intestinal immune modulation. Through downregulating SGLT1, 0GLUT2, somatostatin, and DDP-4 expression, FACE significantly inhibited glucose absorption and contributed to improved glycemic control. Furthermore, FACE enhanced GLP-1 secretion, which is crucial for insulin regulation, appetite control, and metabolic homeostasis. Notably, its immunomodulatory properties (increased T cellular differentiation, plus LL-37, defensin, IL-4, and IL-13 secretion) suggest that FACE strengthens the intestinal barrier and promotes immune defense. Overall, the industrial applicability of FACE is promising, as fermentation significantly enhances its butyric acid concentrations, supporting utilization in functional foods or therapeutic supplements for metabolic disorders. Future studies should focus on clinical validation and formulation optimization to maximize the efficacy of FACE in managing type 2 diabetes and related conditions.

## Data Availability

Data are contained within the article and Supplementary Materials.

## Supplementary Materials

The following supporting information can be downloaded at: https://www.mdpi.com/article/10.3390/cimb47040294/s1.

## Abbreviations

FACE	Fermented *Aralia cordata* extract
ACE	*Aralia cordata* extract
SGLT1	sodium-glucose co-transporter 1
GLUT2	glucose transporter 2
GLP-1	glucagon-like peptide-1
SCFA	short-chain fatty acid
LL-37	cathelicidin antimicrobial peptide
DPP-4	dipeptidyl peptidase-4
T-bet	T-box transcription factor TBX21
GATA3	GATA binding protein 3
Th1	T helper type 1 cells
Th2	T helper type 2 cells
IL-4	interleukin-4
IL-13	interleukin-13
IFN-γ	interferon-gamma
AMPK-γ	AMP-activated protein kinase gamma
