# Supplementary figures and images for: Enhanced Bioactivity of Fermented Aralia cordata Extract for Glucose and Immune Modulation
Source: Curr Issues Mol Biol. 2025 Apr 21;47(4):294. doi: 10.3390/cimb47040294 (PMC12026255; doi:10.3390/cimb47040294)

Figure S1. Treating Dosages of ACE

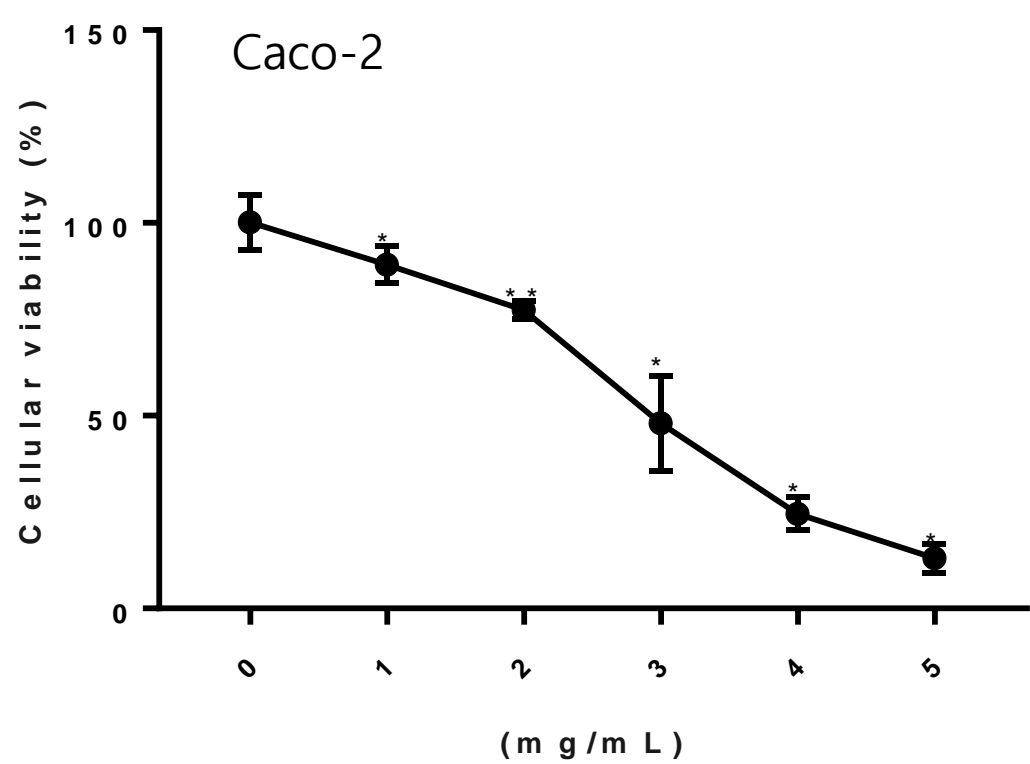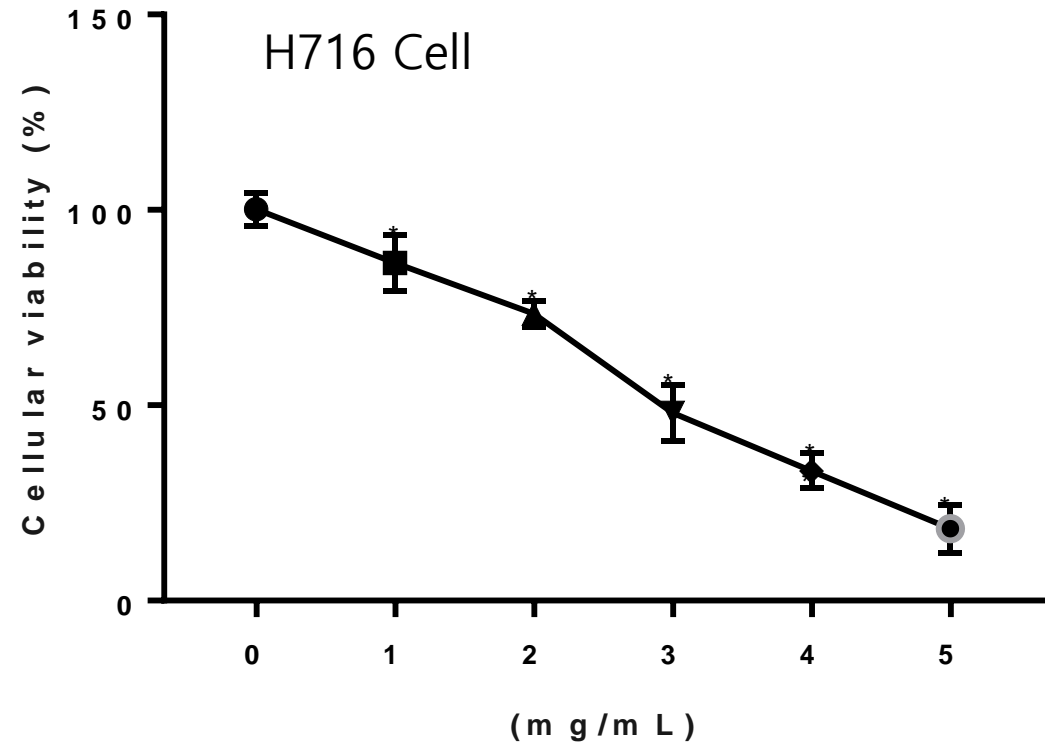

Supplement: Supplementary file 1 [file cimb-47-00294-s001.zip › cimb-3576779-supplementary.pdf]
